# The Rockefeller Foundation and Evidence-based Psychiatry, 1920-1950

**DOI:** 10.1192/j.eurpsy.2025.2385

**Published:** 2025-08-26

**Authors:** J. Wallace

**Affiliations:** 1 Department of Continuing Education, University of Oxford, Oxford, United Kingdom

## Abstract

**Introduction:**

By 1920, the Rockefeller Foundation had set out to systematically improve medical education and clinical practice. This wealthy, formidable agent for change initially focused on surveying the state of medical education world-wide and then targeting individual research institutes and universities with funding aimed at improving their facilities.

**Objectives:**

The aim was to increase understanding of how the Foundation’s focus on improving medical education and practice changed over time.

**Methods:**

The Rockefeller Foundation archive documents the philanthropic activities of the Foundation. Employing a social-history methodology, the primary sources utilised involved officer diaries, including that of Alan Gregg who directed support for medical research and education globally throughout the 1930s and 1940s. Also examined were contemporary journals such as *The British Medical Journal,* the *New England Journal of Medicine,* and *The Lancet;* contemporary newspapers such as the *New York Times*, *The Illustrated London News,* and the *Times of India,* among others. The evidence from the diaries was compared with that of journals, newspapers, and other primary sources.

**Results:**

Initially the Foundation employed a disease-control model aimed at eradicating hookworm, malaria, and yellow fever. However, over time the Foundation changed its focus and developed a special interest in building the discipline of psychiatry. Gregg came to firmly believe that the funding of mental health research and teaching should be given the same resources as any other branch of medicine. Gregg supported initiatives to include psychiatry in standard medical school curricula and he also directed Foundation funding toward individual researchers in the field of mental health. He eventually came to serve as an advisor to the National Institute of Mental Health and the psychiatry section of the Department of Veterans Affairs.

**Conclusions:**

The focus of the Rockefeller Foundation changed over time. Using meticulous planning, the Foundation moved from a laboratory-based, disease-eradication model to developing a deep commitment to promoting scientific psychiatry internationally.

**Disclosure of Interest:**

None Declared

